# Ventricular apical wall rupture and ventricular aneurysm formation concurrent with ventricular septal dissection and rupture due to ST-segment elevation myocardial infarction: a case report

**DOI:** 10.1186/s12872-024-03879-y

**Published:** 2024-04-23

**Authors:** Qianqian Wang, Jingwei Zhou

**Affiliations:** 1 The Affiliated Hospital of Qingdao University, Qingdao, 266000 Shandong China; 2 16, Jiangsu Road, Shinan Disrict, Qiangdao, 266000 Shandong China

**Keywords:** Acute myocardial infarction, Mechanical complications, Bedside echocardiography

## Abstract

The most common mechanical complications of acute myocardial infarction include free-wall rupture, ventricular septal rupture (VSR), papillary muscle rupture and pseudoaneurysm. It is rare for a patient to experience more than one mechanical complication simultaneously. Here, we present a case of ST-segment elevation myocardial infarction (STEMI) complicated with three mechanical complications, including ventricular apical wall rupture, ventricular aneurysm formation and ventricular septal dissection (VSD) with VSR. Cardiac auscultation revealed rhythmic S1 and S2 with a grade 3 holosystolic murmur at the left sternal border. Electrocardiogram indicated anterior ventricular STEMI. Serological tests showed a significant elevated troponin I. Bedside echocardiography revealed ventricular apical wall rupture, apical left ventricle aneurysm and VSD with VSR near the apex. This case demonstrates that several rare mechanical complications can occur simultaneously secondary to STEMI and highlights the importance of bedside echocardiography in the early diagnosis of mechanical complications.

**Supplementary Information:**

The online version contains supplementary material available at 10.1186/s12872-024-03879-y.

## Introduction

Left ventricular free-wall rupture (FWR), ventricular septal rupture (VSR), papillary muscle rupture (PMR), pseudoaneurysm, and true aneurysm are mechanical complications of acute myocardial infarction (AMI). With the development of thrombolytic therapy and primary percutaneous coronary intervention (PCI), the incidence of these complications has decreased to less than 0.1% [1]. However, the occurrence of mechanical complications secondary to ST-segment elevation myocardial infarction (STEMI) is still catastrophic. Left ventricular rupture is a common mechanical complication in AMI patients. Risk factors include no prior history of angina or myocardial infarction, ST-segment elevation in the initial electrocardiogram (ECG), peak creatine kinase isoenzyme MB (CK-MB) above 150 IU/L, female sex, age more than 70 years old, anterior location, transmural infarction and first infarction [2]. VSR is a fatal mechanical complication after AMI that usually requires emergency surgical repair. The 1-month mortality rate of VSR exceeds 50%, and the 3-month survival rate does not exceed 10% without effective treatment [3]. The formation of ventricular aneurysms involves a full-thickness infarct that has been replaced by fibrous tissue. Approximately 85% of the true left ventricular aneurysm (LVA) is located at the apical and anteroseptal walls, while only 5–10% is located at the inferior-posterior or lateral walls, which can be explained by the fact that the apex has only three layers of muscle compared to the four layers at the base [4]. The occurrence of ventricular septal dissection (VSD) is extremely rare after AMI, and only a few cases have been reported in the literature. The most common cause of VSD is right sinus of Valsalva aneurysm [5], and other causes include infective endocarditis, cardiac surgeries, and trauma. The simultaneous occurrence of two or more mechanical complications in one AMI patient is extremely rare. Here, we report a case of mechanical complications of ventricular apical wall rupture, LVA and VSD with VSR after anterior ventricular STEMI.

## Case presentation

**Fig. 1:**
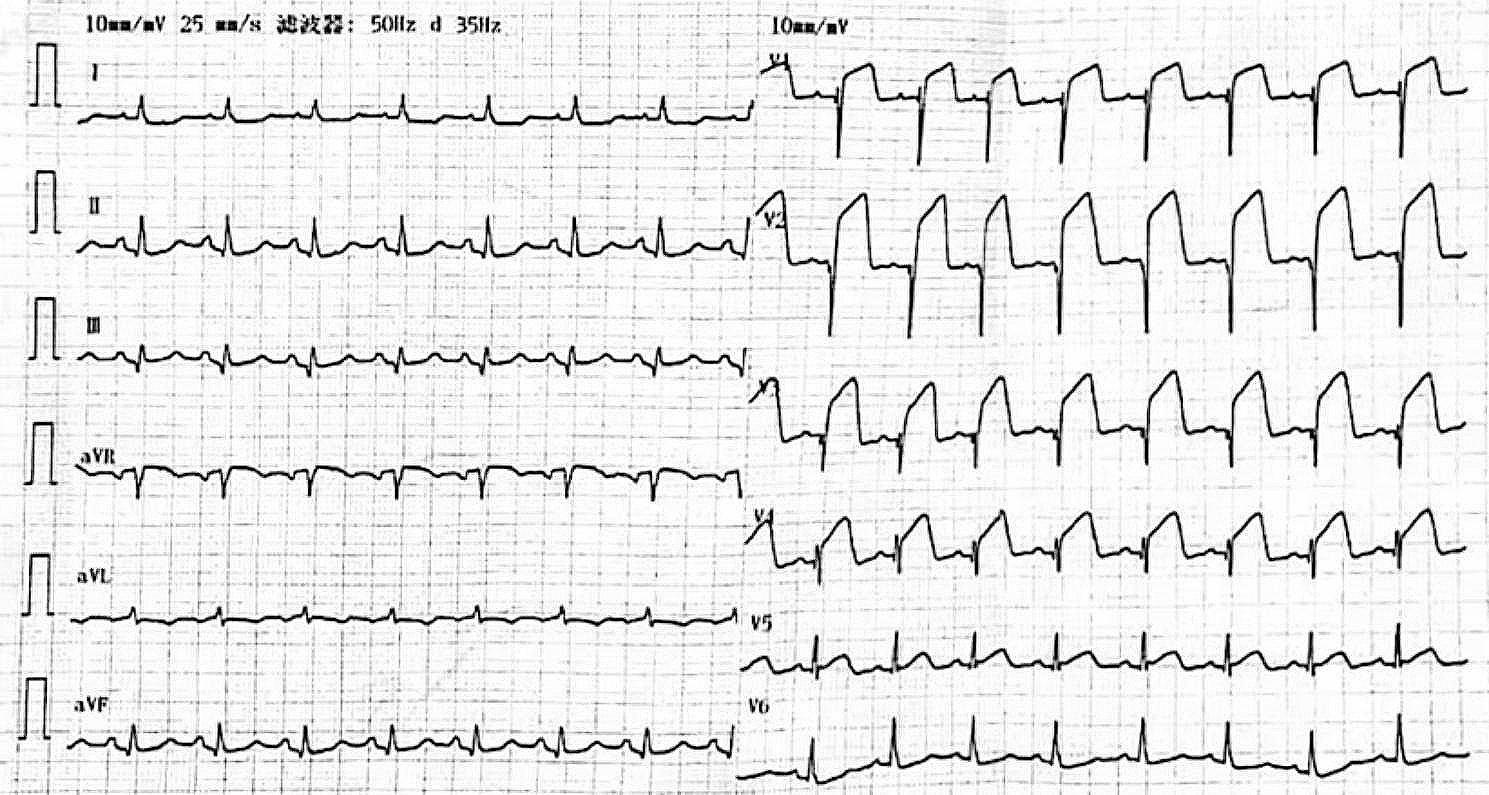
Twelve-leads ECG showed normal sinus rhythm, ST-segment elevation in leads V1-V4 and ST-segment depression in leads I and aVL.

**Fig. 2:**
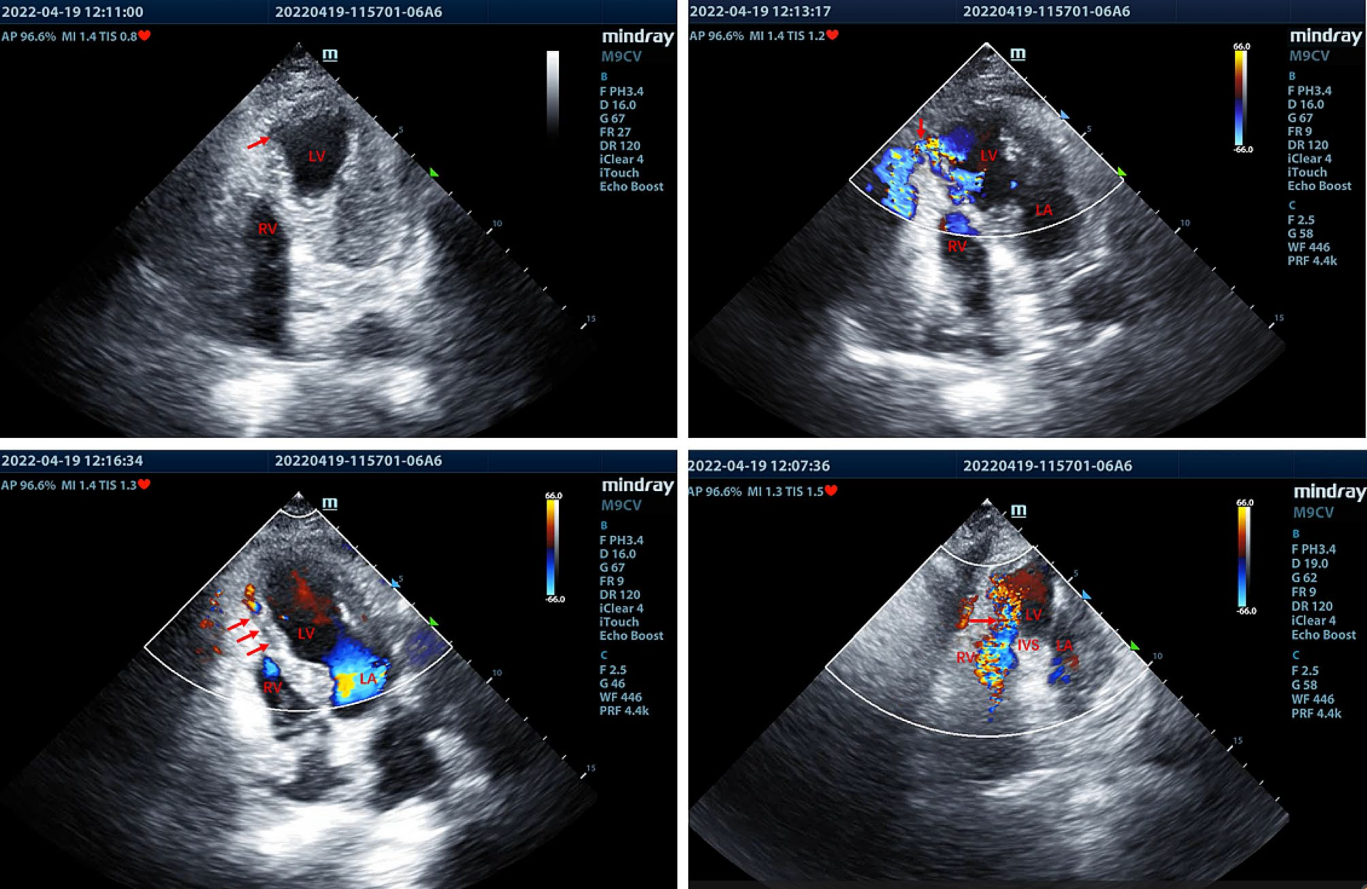
Bedside transthoracic echocardiography revealed ventricular aneurysm formation at the apex of the left ventricle (A) (red arrow), ventricular apical wall rupture (B) (red arrow) and ventricular septal myocardial dissection (C) (red arrow) and interventricular septum (IVS) rupture (D) (red arrow)

A 79-year-old woman with a history of coronary artery disease was admitted to the emergency department in our hospital with chest pain for 7 days. Two days ago, the ECG of the patient was normal in the local hospital. On admission, the ECG showed elevated ST segments in leads V1-V4 and ST-segment depression in leads I and aVL (Fig. 1). The patient was primarily diagnosed with anterior ventricular STEMI. On arrival at the emergency room, the patient had hypotension (90/64 mmHg), and the heart rate was 97 beats per minute. The body temperature was normal (36.8 °C), and the respiratory rate was 20 beats per minute. The respiratory sounds of both lungs were clear, the heart rhythm was regular, and a grade 3 holosystolic murmur was heard at the left sternal border. No edema of the lower extremities was noted. The laboratory analysis showed dramatically elevated CK-MB, troponin I (TNI) and myoglobin, which were 124.6 ng/mL (0-3.6 ng/mL), 19.806 ng/mL (0-0.056 ng/mL) and 2035 ng/mL (10–92 ng/mL), respectively. The D-dimer level was 1660 ng/mL (0-500 ng/mL). The N-terminal pro-B type natriuretic peptide (NT-proBNP) was 5468 pg/mL (0-450 pg/mL), and aspartate transaminase (AST) was 169 U/L (0–45 U/L). The detailed laboratory tests results are summarized in **Supplementary Table 1**. Then, bedside echocardiography was performed, which demonstrated abnormal segmental motion of the left ventricular wall. The left ventricular myocardial motion weakened, and the left ventricular ejection fraction decreased to 40%. A ventricular aneurysm was formed at the apex of the left ventricle with a range of approximately 3.1 × 1.5 cm (Fig. 2A, **Video 1–3**). In addition, it revealed a rupture of the apical ventricular wall with a size of approximately 0.3–0.4 cm and a width of approximately 0.2–0.3 cm, which broke into the pericardial cavity (Fig. 2B, **Video 1–3**). Moreover, a ventricular septal myocardial dissection was approximately 0.1–0.2 cm with a length of approximately 1.8 cm, which ruptured into the right ventricular cavity (Fig. 2C-D, **Video 4–6**). Small to moderate pericardial effusion was also detected.

The patient presented with the symptom of angina, the ECG showed STEMI in the anterior ventricle, and the myocardial enzymes were significantly elevated. Several mechanical complications were detected by bedside echocardiography. Therefore, the final diagnosis was STEMI complicated with ventricular apical wall rupture, LVA and VSD with VSR.

As soon as STEMI was diagnosed, the patient was immediately given a load of aspirin and clopidogrel, along with rosuvastatin. The colloidal solution was administered intravenously to expand blood volume. Coronary angiography was recommended to evaluate the vascular lesions. Surgical repair was the treatment of choice for this patient. However, considering the high risks of surgery, the patient and her family refused further surgical operation or coronary angiography. Approximately 4 h later, the patient refused further treatment and was discharged. Unfortunately, the patient died at home 1 week later. The timeline of the patient from admission to last follow-up was summarized in Table 1.

**Table 1:** The timeline of the patient from admission to last follow-up

Two days before admission	The 79-year-old woman presented with chest pain for 5 days while the ECG was normal in the local hospital.
On admission	She still presented with chest pain and the ECG showed STEMI in the anterior ventricle.
Examination at admission	The patient had hypotension (90/64 mmHg), and the heart rate was 97 beats per minute. The heart rhythm was regular, and a grade 3 holosystolic murmur was heard at the left sternal border.
Laboratory tests after admission	CK-MB was 124.6 ng/mL (0-3.6 ng/mL) TNI was 19.806 ng/mL (0-0.056 ng/mL) Myoglobin was 2035 ng/mL (10–92 ng/mL) D-dimer was 1660 ng/mL (0-500 ng/mL) NT-proBNP was 5468 pg/mL (0-450 pg/mL)
Treatment after primary diagnosis	Aspirin 300 mg, clopidogrel mg, and rosuvastatin 10 mg were taken orally. The colloidal solution was given intravenously to expand blood volume.
Bedside echocardiography	A ventricular aneurysm was formed at the apex of the left ventricle with a range of approximately 3.1 × 1.5 cm. A rupture of the apical ventricular wall was with a size of approximately 0.3–0.4 cm and a width of approximately 0.2–0.3 cm. A ventricular septal myocardial dissection was approximately 0.1–0.2 cm with a length of approximately 1.8 cm, which ruptured into the right ventricular cavity.
Four hours after admission	The patient and her family refused further surgical operation or coronary angiography or further treatment and was discharged.
After 1 week	The patient died at home.

ECG: electrocardiogram; STEMI: ST-segment elevation myocardial infarction; CK-MB: creatine kinase isoenzyme MB; TNI: troponin I; NT-proBNP: pro-B type natriuretic peptide

## Discussion

The incidence of mechanical complications after AMI has significantly decreased in the era of reperfusion, however, the mortality rate remains high. Simultaneous occurrence of two or more mechanical complications after AMI is extremely rare. Only 6 cases of AMI complicated with two mechanical complications have been reported so far (Table 2). Our case is the first to report three mechanical complications secondary to STEMI, including ventricular apical wall rupture, LVA and VSD with VSR.

**Table 2:** Cases of AMI concurrent with two mechanical complications reported in recent years

Mechanical Complications	Patient’s age (years) and sex	Treatments	Outcomes
LVA and VSR [1]	83; Female	Surgical repair	Survival
LVA and VSR [2]	70; Male	Surgical repair	Survival
VSR and VSD [3]	72; Male	Surgical repair	Died
LVA and VSR [4]	65; Female	Surgical repair	Survival
VSR and free wall rupture [5]	74; Female	Transcatheter closure	Died
VSR and VSD [6]	65; Female	Transcatheter closure	Survival

LVA: Left ventricular aneurysm; VSR: Ventricular septal rupture; VSD: Ventricular septal dissection

**Reference**

[1] Sato H, Naraoka S. Left Ventricular Patch Plasty for Post-infarct Ventricular True Aneurysm with Ventricular Septal Perforation; Report of a Case. Kyobu Geka. 2016;69(11):956–958

[2] Mizumoto M, Uchida T, Hamasaki A, et al. Patch Closure of Posterior Type Ventricular Septal Perforation with Posterior Left Ventricular Aneurysm through Right Ventricular Incision. Kyobu Geka. 2020;73(2):94–98

[3] Pires de Morais G, Paulo N, Vieira MS, et al. Complex ventricular septal rupture with dissection of the right ventricular wall in ischemic context. Echocardiography. 2012;29(5):E112-114

[4] Zhang P, Pang X, Yu D, Zhang Y. Concurrent true inferoposterior left ventricular aneurysm and ventricular septal rupture secondary to inferior myocardial infarction: a case report. Eur Heart J Case Rep. 2018;2(4):yty136

[5] Xenogiannis I, Chavez I, Hall AB, Brilakis ES. Interventricular septum and free wall rupture in a patient with non-ST-segment elevation myocardial infarction: A lethal combination. Hellenic J Cardiol. 2019;60(5):341–343

[6] Zhong X, Zhou G, Huan Z, et al. Small septal vessel occlusion results in big damage: ventricular septal dissection and rupture. Eur Heart J. 2018;39(26):2506–2507

The post-infarction ventricular aneurysms may be a true aneurysm or a pseudoaneurysms. True LVA forms when ventricular pressure causes the expansion of infarcted and relatively fragile area, which occurs within days to weeks following STEMI. Left ventricular pseudoaneurysm occurs when a rupture of the ventricular free wall is contained by overlying adherent pericardium. The most common location of left ventricular pseudoaneurysm is the posterior wall, followed by the lateral, apical and inferior wall. The neck is wide in true aneurysm while narrow in pseudoaneurysm, which is considered the main difference. Transthoracic echocardiography has high diagnostic performance for LVA, with sensitivity and specificity exceeding 90% [6]. Surgical resection is considered the most appropriate treatment for left ventricular pseudoaneurysm because of the high risk of rupture, while for true LVA, medical treatment is mainly recommended. According to echocardiography, the ventricular aneurysm in this case was diagnosed as a true LVA, which was located at the apex of the left ventricle and was a wide-neck aneurysm.

In our case, bedside echocardiography showed dissection of ventricular septal myocardium, which eventually ruptured into the right ventricular cavity. Thus, the VSR of this patient was more likely the result of VSD, rather than two independent complications. The pathophysiology of VSD secondary to AMI remains unclear. Possible hypotheses include interruption of blood flow in the interventricular septal perforator artery, right sinus of Valsalva aneurysm, helical myocardial muscular band and occlusion of small interventricular septal vessels [5]. In addition to the symptoms of AMI, the typical symptoms of VSD include hypotension and harsh systolic murmurs. VSD can be diagnosed by transthoracic echocardiography, which generally shows an interventricular false cavity that is connected to the left ventricle or right ventricle [5]. VSR is almost always associated with major coronary artery lesions. Surgical repair is the main recommended guideline for patients with VSR after AMI. With the high success rate, transcatheter closure of VSR has become a valuable alternative to surgical repair, which can immediately reduce shunts and prevent hemodynamic deterioration. However, the optimal therapeutic timing for VSR closure is an ongoing debate. Previous studies have shown that mortality is lower when surgeries are delayed for a week than when they are performed immediately [7]. Similarly, transcatheter closure of VSR has been demonstrated to be feasible and effective in the deferred period of the rupture. The high mortality of VSR patients undergoing percutaneous or surgical repair in the acute phase may be related to the infarcted myocardium, which is weak and fragile, and the unstable hemodynamic status in the acute phase after AMI [8]. Female sex and Killip Class III-IV at admission are associated with an increased risk of death in the acute phase of VSR after infarction.

According to the characteristics of echocardiography, the blood flow entered the pericardium through the ventricular rupture was not blow out. Therefore, the ventricular rupture in this case was considered an oozing-type rupture rather than a blowout rupture. The blowout rupture is often associated with haemodynamic instability and/or cardiac arrest. While the oozing-type rupture may be restrained by thrombosis and compliant pericardium, which is not usually associated with cardiogenic shock [9]. In addition, we found that there seems to be a thrombus at the rupture of the left ventricular apex, which restricted the blood flow to some extent (**Supplementary video 1–2**). These may explain why the patient didn’t develop tamponade immediately after the rupture. This patient had several risk factors for mechanical complications of AMI, such as female sex, old age, first attack of AMI, low ejection fraction and unstable hemodynamic status. The ECG showed anterior ventricular STEMI, so we inferred that the lesion was mainly located in the left anterior descending branch coronary artery. However, the limitation of this case is that invasive coronary angiography and coronary CT angiography were not performed, so the responsible vessels could not be verified. Another limitation is that the patient gave up surgical repair, so we can’t determine whether surgical treatment can save this AMI patient with such complex complications.

Stress cardiomyopathy, which is also called Takotsubo cardiomyopathy (TC), is reported in about 2–3% of all troponin-positive suspected acute coronary syndrome (ACS) cases [10]. TC is similar to ACS and is characterized by reversible left ventricular apical ballooning in the absence of angiographically substantial coronary artery stenosis. Patients with TC typically present with acute onset chest pain and/or dyspnea, which are usually associated with acute stress events. In this case, the patient did not have any emotional, physical, or combined trigger prior to ACS. Before the patient came to our hospital, she had suffered from chest pain for 7 days with a normal ECG. The course of the disease was more like a typical progression of angina to AMI, rather than TC characterized by acute onset chest pain. Besides, the laboratory tests showed significantly elevated TNI and mildly increased NT-proBNP. In TC, the TNI is usually moderately increased while the NT-proBNP is markedly increased due to more important degree of acute left ventricular dilation and myocardial stretch. The ECG also did not completely match the typical ECG features of TC, in which ST-segment elevation in precordial leads without reciprocal ST-segment depression in inferior leads. Left ventricular free wall rupture can occur in both AMI and TC, however, apical aneurysm is more common in AMI. Recently, the Takotsubo International Registry proposed a clinical diagnostic score to assess the clinical probability of TC and try to distinguish it from AMI before imaging tests and coronary angiography are performed [11]. When patients with a score of ≥ 50 were diagnosed as TC or with a score of ≤ 31 were diagnosed as ACS, nearly 95% of the patients were correctly diagnosed. In this case the score of the patient was 25. Therefore, we preliminarily excluded TC to some extent. However, according to the diagnostic criteria proposed by the Mayo Clinic, coronary angiography is required to completely rule out TC, which remains a limitation of this case.

All these mechanical complications are life-threatening for AMI patients and required early diagnosis and urgent repair in the published cases [12]. Transthoracic echocardiography has advantages in the detection of STEMI-related compilations including left ventricular thrombi, infarct expansion, true aneurysm formation, post-infarction pericarditis, pericardial effusion and tamponade. This non-invasive method provides convenient estimation of impaired blood supply to myocardial tissue. In addition to anatomical information, the evaluation of ventricular wall motion by real-time echocardiography has important prognostic significance. In particular, the echocardiography is very useful in the identification and localization of mechanical complications after AMI and allow for careful monitoring of patients before, during and after surgical repair. If appropriate interventions are applied, early recognition of the mechanical complications will improve the survival of AMI patients. Therefore, it is hoped that with the improvement of echocardiography in evaluating mechanical complications and the more rapid surgical intervention, future studies will show better outcomes in AMI patients with mechanical complications.

## Conclusion

We presented a case of STEMI complicated with ventricular wall rupture, ventricular aneurysm formation and VSD with VSR for the first time. Physicians need to be aware of the complex mechanical complications of AMI and be more vigilant, especially for patients who present late and are hemodynamically unstable. The presence of a cardiac murmur is an enormous clue that will help make the diagnosis. Bedside echocardiography is valuable in the diagnosis of complications after AMI and has high diagnostic sensitivity and specificity.

### Electronic supplementary material

Below is the link to the electronic supplementary material.

Supplementary Material 1

Supplementary Material 2

Supplementary Material 3

Supplementary Material 4

Supplementary Material 5

Supplementary Material 6

Supplementary Material 7

Supplementary Material 8

Supplementary Material 9

Supplementary Material 10

## Data Availability

The data of this study are available from the corresponding author upon reasonable request.
