# Supplementary material for: Ventricular apical wall rupture and ventricular aneurysm formation concurrent with ventricular septal dissection and rupture due to ST-segment elevation myocardial infarction: a case report
Source: BMC Cardiovasc Disord. 2024 Apr 23;24:222. doi: 10.1186/s12872-024-03879-y (PMC11036618; doi:10.1186/s12872-024-03879-y)
Supplement: Supplementary file 1 — Supplementary Material 1 [file 12872_2024_3879_MOESM1_ESM.docx]

**Supplementary Table 1.** The detailed laboratory test results.

| **Laboratory tests** | **Main test items** | **Results; Reference ranges** |
| --- | --- | --- |
| **Complete blood count** | Count of white blood cells  Count of neutrophils  Proportion of neutrophils  Count of lymphocytes  Proportion of lymphocytes  Count of monocytes  Proportion of monocytes  Hemoglobin  Count of platelets | 19.20×10^9^/L; (3.5-9.5)×10^9^/L  17.65×10^9^/L; (1.8-6.3)×10^9^/L  92.00%; (40-75)%  0.95×10^9^/L; (1.1-3.2)×10^9^/L  4.9%; (20-50)%  0.56×10^9^/L; (0.1-0.6)×10^9^/L  2.9%; (3-10)%  140 g/L; (115-150) g/L  201×10^9^/L;(125-350)×10^9^/L |
| **Emergency**  **hepatic functions** | Albumin  Total bilirubin (TBIL)  Alanine aminotransferase (ALT)  Aspartate aminotransferase (AST) | 45.60 g/L; (40-55) g/L  16.4 umol/L; (3-22) umol/L  37 U/L; (9-52) U/L  169 U/L; (14-36) U/L |
| **Emergency**  **renal functions** | Urea nitrogen  Creatinine | 7.30 mmol/L; (3.1-8.8) mmol/L  88.21umol/L; (31-132) umol/L |
| **Coagulation functions** | Prothrombin time (PT)  International normalized ratio (INR)  Fibrinogen  Activated partial thromboplastin time (APTT)  Thrombin time (TT)  D-dimer | 15.10 s; (10.5-14.5) s  1.19; (0.75-1.15)  2.72 g/L; (2-4) g/L  36.80 s; (28-43.5) s  18.6 s; (14-21) s  1660 ng/mL; (0-500) ng/mL |
| **Cardiac enzymes and NT-proBNP** | Troponin I (TNI)  Creatine kinase isoenzyme MB (CK-MB)  Myoglobin  N-terminal pro-B type natriuretic peptide (NT-proBNP) | 19.806 ng/mL; (0-0.056) ng/mL  124.6 ng/mL; (0-3.6) ng/mL  2035 ng/mL; (10-92) ng/mL  5468 pg/mL; (0-450) pg/mL |
| **Serum electrolytes and blood glucose** | Potassium (K)  Sodium (Na)  Chlorine (Cl)  Carbon dioxide (CO_2_)  Anion gap (AG)  Glucose | 4.40 mmol/L; (3.5-5.5) mmol/L  143.40 mmol/L; (136-146) mmol/L  102.60 mmol/L; (96-108) mmol/L  23.7 mmol/L; (22.0-30.0) mmol/L  17.1 mmol/L; (8-16) mmol/L  14.07 mmol/L; (3.9-6.16) mmol/L |
