# Supplementary material for: Ventricular apical wall rupture and ventricular aneurysm formation concurrent with ventricular septal dissection and rupture due to ST-segment elevation myocardial infarction: a case report
Source: BMC Cardiovasc Disord. 2024 Apr 23;24:222. doi: 10.1186/s12872-024-03879-y (PMC11036618; doi:10.1186/s12872-024-03879-y)
Supplement: Supplementary file 10 — Supplementary Material 10 [file 12872_2024_3879_MOESM10_ESM.docx]

**Video introductions**

**Video 1-Video 3** mainly shows ventricular apical wall rupture and left ventricular aneurysm (LVA).

**Video 1.** During ventricular contraction, the myocardium at the apex of the left ventricle becomes thinner and locally protrudes outward, forming a ventricular aneurysm (upper left). In addition, there is a myocardial discontinuity at the apex of the left ventricle, which ruptures into the pericardium (upper left).

**Video 2** and **Video 3.** These two videos mainly display left ventricle (upper) and right ventricle (below). Video 2 displays the ventricular wall rupture and LVA at the apex of the left ventricle again. In video 3, the blood flow enters pericardial cavity from the left ventricle during ventricular contraction (upper left). The blood flow was not blow out. The blood flow from the left ventricle to right ventricular cavity can also be seen (below).

**Video 4-Video 6** mainly shows ventricular septal dissection (VSD) with ventricular septal rupture (VSR).

**Video 4.** This video mainly shows VSD. During ventricular contraction, blood flow enters the interventricular septum through the left ventricle endocardial rupture, and a channel is formed in the interventricular septum.

**Video 5** and **Video 6.** These two videos mainly display left ventricle (upper) and right ventricle (below). The ventricular septum is discontinuous. During ventricular contraction, blood flow enters the right ventricular cavity from the left ventricle through the rupture of the interventricular septum (below). Meanwhile the blood flow from left ventricle to pericardial cavity can also be seen (upper left).
